# Supplementary material for: Prediction of pharmaceutical and non-pharmaceutical expenditures associated with Diabetes Mellitus type II based on clinical risk
Source: PLoS One. 2024 Jun 4;19(6):e0301860. doi: 10.1371/journal.pone.0301860 (PMC11149868; doi:10.1371/journal.pone.0301860)
Supplement: Attachment — Submitted filename: Response letter to reviewers 22-03-23.docx [file pone.0301860.s001.docx]

PONE-D-22-29157

Prediction of pharmaceutical and non-pharmaceutical expenditures associated with Diabetes Mellitus type II based on clinical risk

PLOS ONE

**Comments to the Author**

Reviewer #1: The context of this study has a high relevance and can potentially be useful for resource planning in hospitals.

However, before publication, several issues must be addressed.

- Firstly, the conclusions of this study are not yet compelling enough for the analysis done. In that sense, I ask you to rewrite that section (as well as the abstract).
  This is important because you mention the relevance of studying the relationship between the input-output variables as a motivation for the study. Tables 3 and 4 are quite interesting and go in line with the initially mentioned matter of the paper. However, there is no actual discussion on this.

R:// We appreciate the comment and have improved the introduction, abstract, and conclusions clarifying the contribution and limitations of the study.

- A Reorganisation of the document must be done. For instance, sections of the discussion clearly belong in the introduction or related work. Figure 1 doesn't need to be segregated from the main text. Related studies and descriptions of the previously used methods are part of the related work, not the discussion. The first part of the manuscript seems to be stitched together without much consideration for the previous methodological work. Then the second part mentions some previous work's results, but the discussion needs to be completed.

R:// We appreciate the comment and have made the following changes:

. we have added Figure 1 to the text.

. we have improved the description of the related work.

. we have reorganized the works presented in the discussion section and we have moved them into the introduction section.

- The lack of reference to important studies in this context shows a lack of a comprehensive review, which leads to some bold claims. For instance, how does this research build upon:
  Awad, Susanne F., et al. "Forecasting the burden of type 2 diabetes mellitus in Qatar to 2050: a novel modeling approach." Diabetes research and clinical practice 137 (2018): 100-108.
  or Khanal, Saval, et al. "Forecasting the amount and cost of medicine to treat type 2 diabetes mellitus in Nepal using knowledge on medicine usage from a developed country." Journal of Pharmaceutical Health Services Research 10.1 (2019): 91-99.
  Please also review the English and eventual translations like "Data transformación" and "Data Pre-Datacasting". Check also the comas and points issue: "US$ 1,125 and US$ 1.234"

R:// We appreciate the comment and have updated the papers described and checked the typos .

- Also, format the tables in the same fashion throughout the text for uniformity.

R:// We have used the same format for tables in the whole document.

- There must be at least a reference to support the choice of performance evaluation. A few graphs on the Descriptive Analysis are a critical addition to better categorize de distributions.

R:// We have added a reference of the metrics used.

- For the above-mentioned reasons, I believe that this manuscript has some potential but needs a comprehensive amount of work before publication.

R:// We appreciate this comment for letting us improve our paper.

Reviewer #2: Summary

The goal of this work is to assess the effectiveness of different machine learning models in estimating pharmaceutical and non-pharmaceutical expenditures associated with Diabetes, considering the comorbidities of the patient. The models considered were Linear Regression, Lasso Regression, and Neural Networks. The results showed greater accuracy in predicting pharmaceutical and non-pharmaceutical expenditures through Neural Networks.

R:// We appreciate this comment for letting us improve our paper.

Minor comments

- The submitted document had two different formatting types. In future submissions, try to uniformize the font and size used.

R:// We appreciate this suggestion, we have checked and changed the entire document using the same type of font and size.

- In table 3, the results of the linear regression display the estimation of the intercept (constant), the dummy variable for Man (M), and the dummy variable for Woman (W). This table should be reviewed, since in the linear model, including both categories and the intercept would mean we are violating the absence of perfect collinearity assumption, which means we could not estimate the model through OLS.

R:// We appreciate this comment, we have updated the tables clarifying the changes made to guarantee the assumption of the OLS model.

- More details on the methods used should be added in the Methodology and Results section. The results could also be complemented with some plots.

Major comments

Overall, this work is well written. However, it falls short concerning the implemented methodology. The result that Neural Networks are more efficient is expected. These algorithms have been used before to predict not only the risk/probability of a specific disease but also medical costs. The major contribution is the inclusion of Charle's Index as an attribute in the models. Thus, this work should be enriched with other methods, besides the ones implemented. For example, cluster the individuals based on the Charle's Index and then apply the models to each cluster or consider the panel data structure (since in this work each time that a patient is admitted to the hospital is treated as a single source of data, therefore patients with multiple registrations to the hospital in different periods are analyzed as different record).

R:// We agree with reviewers, nevertheless, we have a hard constraint given the database that doesn´t allow us to split data into clusters because we don´t have enough records and we’re not able to get more of them.

Reviewer #3:

- The authors need to explain in depth all the used variables in the model. Note that using the same variable as independent and dependent invalidates any accuracy of the model, hence the authors must strongly justify why they used hospitalization costs as independent variable (which include pharmaceutical and non-pharmaceutical expenditures), and as dependent variable also expenditure (composed of pharmaceutical and non-pharmaceutical expenditures). This intorduces a high risk of biased resuts.

R:// We agree with the reviewer, however, we have clarified in the text that the independent variables calculated are related to the number of medicine consumption (pharmaceutical and non-pharmaceuticals) but not to the expenditures.

- The authors must also rewrite the introduction and include the study contributions, as well in the methodology, it is fundamental to support theoretically each used item/variable included in the model. It is of most importance the conclusions contain implications and limitations of the study.

R:// We agree with the reviewer, therefore we have improved the introduction including related works and we have included the implications and limitations of the study.

Kind Regards,

Javier González Rodriguez

Correspondence Author
