# Supplementary material for: Prediction of pharmaceutical and non-pharmaceutical expenditures associated with Diabetes Mellitus type II based on clinical risk
Source: PLoS One. 2024 Jun 4;19(6):e0301860. doi: 10.1371/journal.pone.0301860 (PMC11149868; doi:10.1371/journal.pone.0301860)
Supplement: Attachment — Submitted filename: Response letter to reviewers Last Revers Answer.docx [file pone.0301860.s002.docx]

PONE-D-22-29157

Prediction of pharmaceutical and non-pharmaceutical expenditures associated with Diabetes Mellitus type II based on clinical risk

PLOS ONE

We are very proud to send our improved version of the article, in which the improved versions are shown. The article has the changes indicated in green.

Below are the changes in response to reviewer comments.

**Comments to the Author**

Reviewer #4: The authors have successfully defended all comments of previous reviewers. They have actually improved the manuscript according to the suggestions. Hence, the current form of the paper may be accepted.

They may cite some related and recent papers in the final manuscript:

https://doi.org/10.1007/s11042-020-10242-8; https://doi.org/10.1007/978-981-13-1280-9

R:// we have included a paragraph in the text including the recent papers related with our topic.

Kind Regards,

Javier González Rodriguez

Correspondence Author
